# Seasonal purchase of antihistamines and ovarian cancer risk in the Cancer Loyalty Card Study (CLOCS): results from an observational case-control study

**DOI:** 10.23889/ijpds.v8i3.2292

**Published:** 2023-09-11

**Authors:** Hannah Brewer, Qianhui Jiang, Sudha Sundar, Yasemin Hirst, James Flanagan

**Affiliations:** 1 Division of Cancer, Department of Surgery and Cancer, Imperial College London; 2 Institute of Cancer and Genomic Sciences, University of Birmingham; 3 Department of Behavioural Science and Health, University College London

## Introduction & Background

Antihistamine use has been associated with a reduction in ovarian cancer incidence. Herein, we investigate antihistamine exposure in relation to ovarian cancer risk using a novel data resource by examining purchase histories from retailer loyalty card data.

## Objectives & Approach

Participants from the Cancer Loyalty Card Study (CLOCS) included ovarian cancer patients (cases, n=153) and women without a diagnosis of ovarian cancer (controls, n=120). Up to 6 years of purchase history was retrieved from two participating high street retailers from 2014-2022. Logistic regression was used to estimate the odds ratio (OR) and 95% confidence intervals for ovarian cancer associated with antihistamine purchases, adjusting for confounders. The association was stratified by season of purchase, age, histology, and family history.

## Relevance to Digital Footprints

This study is one of the first to utilise transaction data from high street retailers to investigate associations with cancer risk, based on what participants are buying.

## Results

Ever purchasing antihistamines was not significantly associated with ovarian cancer overall in this small study (OR=0.68 (0.39-1.19)). However, antihistamine purchases were significantly associated with reduced ovarian cancer risk when purchased only in spring and/or summer (OR=0.37 (0.17-0.82)) and in non-serous ovarian cancer (OR=0.41 (0.18-0.93)) in stratified analyses.

## Conclusions & Implications

Antihistamine purchase is associated with reduced ovarian cancer risk when purchased seasonally. However, larger studies are required to understand the mechanisms of reduced ovarian cancer risk related to seasonal purchases of antihistamines and allergies.

